# Supplementary material for: Learning curve and surgical outcome of robotic assisted colorectal surgery with ERAS program
Source: Sci Rep. 2022 Nov 29;12:20566. doi: 10.1038/s41598-022-24665-w (PMC9709162; doi:10.1038/s41598-022-24665-w)
Supplement: Supplementary file 2 — Supplementary Information 2. [file 41598_2022_24665_MOESM2_ESM.zip › RAL Raw data-Ñ[▒K/073/OP_ñw╝╨░O▒Kñσ.pdf]

正式摘要(電子簽章：000000214282982)

請選擇...reporter

臺中榮民總醫院

Operation Note

[redacted]

- 手術日期：2021/04/09
- 手術醫師：VS 蔣鋒帆，CR 林陳彥，CR 江世偉，R 陳威遠
- Anesthesia：GE
- Position：Supine
- Aseptic agents：Betadine Aqua，Zephnan

Diagnosis

- Pre-operative diagnosis : Malignant tumor of A-colon
- Post-operative diagnosis : s/p robotic right hemicolectomy

Surgical Indication

- For removing the primary tumor

Operation

- 1. Robotic (XI) assisted right hemicolectomy
  2. Intraoperative colonoscopy : (-)
  3. Ileo-colic artery, right colic artery and right branch of middle colic artery were all ligated via robotic endoclips.
  4. Take down from the hepatic flexure : (+)
  5. OpenGIA (80-3.8) X2 were applied to complete the side to side anastomosis
  6. Cut margin: proximal (ileum) 5 cm, (colon) 3 cm, distal 5 cm.
  7. No drainage tube was inserted
  8. ADHESION BARRIER FILM was placed below the fascia of the para-umbilicus wound

Finding

- 1. A 3 X 3 cm, ulcerative tumor over middle A-colon area. A suspicious enlarged lymph nodes at ileocolic area. It was resected for sampling.
  2. No other gross tumor was found.
  3. There was no visible liver metastasis or visible peritoneal seeding.

Procedure

- 1. General endotracheal anesthesia was induced and then the patient was placed in supine position.

  2. Preoperative antibiotics were given. A Foley catheter and nasogastric tube were placed.
  3. The abdomen was prepped and draped in standard sterile fashion.
  4. A small horizontal incision was made over the para-umbilicus (left) area and a 8 mm trocar was inserted into the peritoneal cavity by open method. Peumoperitoneum was established with CO2 inflation.
  5. Laparoscopy was inserted and then other three 8mm trocars were inserted over LLQ (X2) and LUQ (X1) of abdomen. The assist-port 5mm troca was inserted on LLQ. The operative findings were mentioned as above.
  7. Omentum was freed from the transverse colon and lesser sac was entered.
  8. The left branch of middle colic artery was identifired and was later ligated with endoclips.
  9. The dissection was performed carefully toward hepatic flexure site until the proximal T-colon was partially mobile.
  10. The small bowel was inspected and retracted to downward by grasper. The peritoneum overlying the mesentery was then scored with electrocautery and the ileocolic artery and the adjacent vein

- were identified, ligated with endoclips, and transected via the electrical cautery instruement of XI system
  11. A suspicious lymphnode was noted near ileocolic artery area. It was resected for sampling.
  12. After the vessel were all under controlled, the colon was freed from its peritoneal attachments along the line of Toldt from the cecum to the hepatic flexure.
  13. Hemostasis was checked in the operative field. Cecum, ascending colon and hepatic flexure were mobilized and then laparoscope was removed.
  14. The incision was extended over left para-umbilical area.
  15. The ascending colon and the T-colon was delivered from the incision. The points of transection were selected proximally and distally.
  16. Side to side anastomosis was performed via firing two staples of OpenGIA (80-3.8). Interrupted 3-0 silk Lambert sutures were used to re-inforcement at outer layer.
  17. The anastomosis was checked and found to be intact and widely patent.
  18. The small bowel and colon was put back into the abdomen. Pneumoperitoneum was established again.
  19. Possible bleeder was examed again. The orientation of the small bowel was checked thoroughly.
  20. ADHESION BARRIER FILM was placed below the fascia of the para-umbilicus wound
  21. The fascia of the extended wound was closed with continous sutures via Num.1 Vicryl. (2 layers.) The fascia of the remaing 8mm trochars were all closed with interrupted method via 1-0 Vicryl.
  22. The skin wound was sutured with interrupted method via 3-O Vicryl, then covered with Surgicel and Medipore.
  23. The patient tolerated the procedure well and was brought to the POR in stable condition.
- Estimated blood loss: minimum
- 器械數量: 正確
- 紗布數量: 正確

Image

- PAINT
  - abdomen

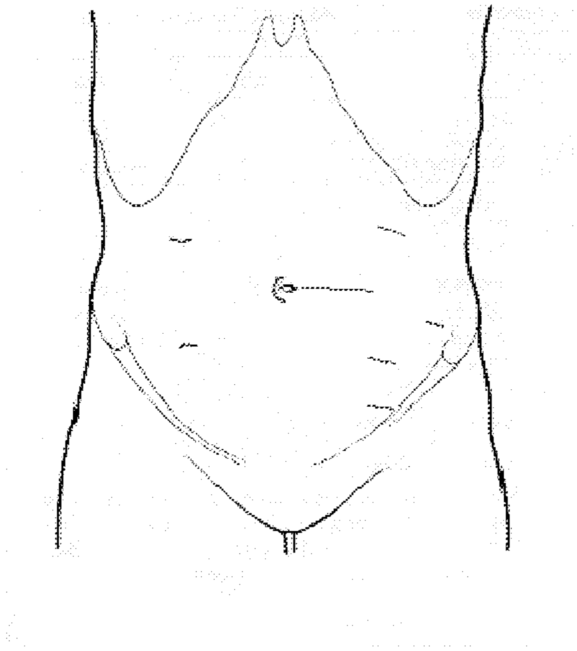

  - colon & rectum

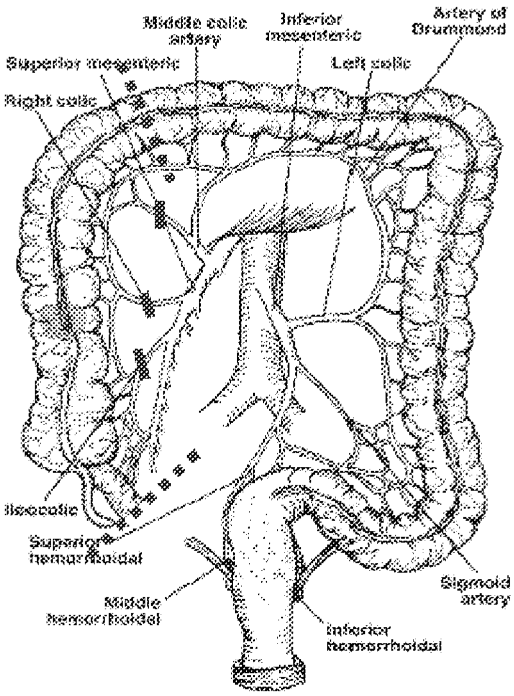

- The resected specimen (A-colon and terminal ileum)

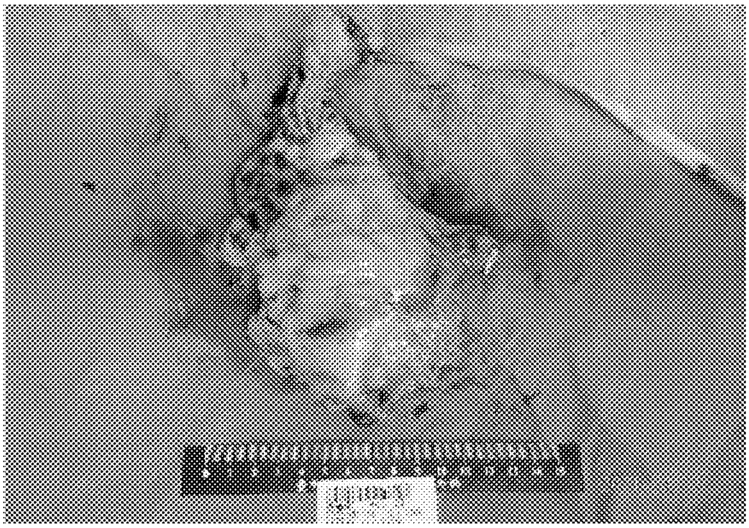

The tumor could be clearly seen

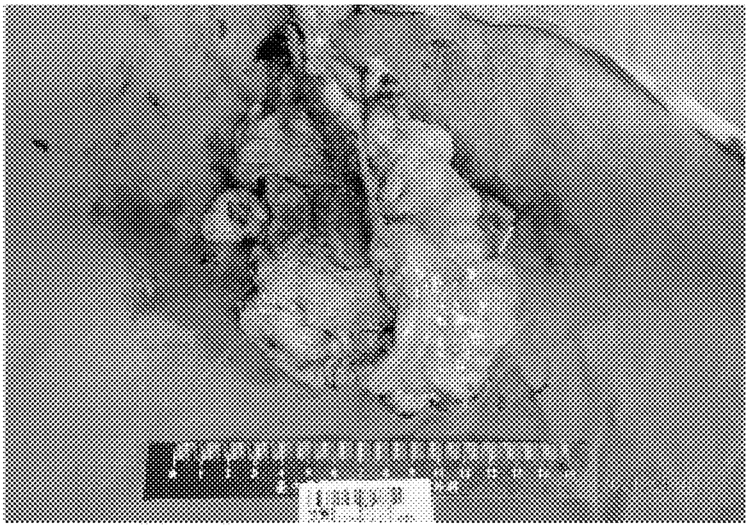

主任 2062F蔣鋒帆 / 醫師 2301H林陳彥

正式摘要(電子簽章：**000000214282982)**

請選擇...reporter

doctorRank:B
